# *Drosophila* Mcm10 Is Required for DNA Replication and Differentiation in the Compound Eye

**DOI:** 10.1371/journal.pone.0093450

**Published:** 2014-03-31

**Authors:** Nicole Vo, Ayano Taga, Yasuhiro Inaba, Hideki Yoshida, Sue Cotterill, Masamitsu Yamaguchi

**Affiliations:** 1 Department of Applied Biology, Kyoto Institute of Technology, Kyoto, Japan; 2 Insect Biomedical Research Center, Kyoto Institute of Technology, Kyoto, Japan; 3 Department of Basic Medical Sciences, St Georges University of London, London, United Kingdom; University of Minnesota, United States of America

## Abstract

Mini chromosome maintenance 10 (Mcm10) is an essential protein, which is conserved from *S. cerevisiae* to *Drosophila* and human, and is required for the initiation of DNA replication. Knockdown of *Drosophila Mcm10* (*dMcm10)* by RNA interference in eye imaginal discs induces abnormal eye morphology (rough eye phenotype), and the number of ommatidia is decreased in adult eyes. We also observed a delay in the S phase and M phase in eye discs of *dMcm10* knockdown fly lines. These results show important roles for dMcm10 in the progression of S and M phases. Furthermore, genome damage and apoptosis were induced by *dMcm10* knockdown in eye imaginal discs. Surprisingly, when we used *deadpan-lacZ* and *klingon-lacZ* enhancer trap lines to monitor the photoreceptor cells in eye discs, knockdown of *dMcm10* by the *GMR*-GAL4 driver reduced the signals of R7 photoreceptor cells. These data suggest an involvement of dMcm10 in R7 cell differentiation. This involvement appears to be independent of the apoptosis induced by *dMcm10* knockdown. Together, these results suggest that *dMcm10* knockdown has an effect on DNA replication and R7 cell differentiation.

## Introduction

Minichromosome maintenance protein 10 (Mcm10) is needed for DNA replication in eukaryotes [1]–[6]. Several previous studies in yeast and *Xenopus laevis* suggested that Mcm10 is recruited to chromatin after origin licensing but before the initiation step of DNA replication [1]–[3]. However, recent *in vitro* studies in yeast have shown that the recruitment of Mcm10 to the origin occurs after the assembly of the Cdc45-Mcm2-7-GINS complex (CMG) [4], [5]. Consistent with this latter model, two other *in vivo* studies demonstrated that Cdc45 loading onto chromatin was independent of Mcm10 [2], [7].

A direct or indirect role of Mcm10 in remodeling the CMG helicase to promote DNA unwinding has been debated for years. Some groups have suggested that Mcm10 works as an active helicase activator [8], while others have demonstrated its role as a facilitator of single stranded DNA (ssDNA) binding [9], [10]. In the former model, Mcm10 may act to release one DNA strand from the core of CMG complex at the origin, and then assist the translocase activity in the 3′ to 5′ direction resulting in origin unwinding and replication initiation. Alternatively, in the latter model, after the active CMG complex unwinds the origin of replication, Mcm10 may stabilize the formation of ssDNA via its ssDNA-binding domain in order to initiate DNA replication.

Mcm10 has three functional domains: an N-terminal domain (NTD), the internal domain (ID) and a metazoan-specific C-terminal domain (CTD). The N-terminal domain is not highly conserved across species. This domain was reported to be responsible for self-association of Mcm10 protein [11] and studies in *Xenopus* showed that the NTD has a role in homodimerization [12]. This might be involved in the association of Mcm10 multimers, which has been suggested to be important to facilitate multiple interactions with other replication factors and DNA [2], [13]. In contrast, the internal domain is highly conserved among species. This domain consists of an oligonucleotide binding fold (OB-fold) which forms a typical DNA-binding cleft [9], [14]. The OB-fold comprises three important regions. The first one is an Hsp10-like domain, a conserved hydrophobic patch, where the interaction with DNA polymerase α (polα) occurs [9], [14], [15]. The second region, the PCNA-interacting peptide (PIP) box motif, overlaps partially with the DNA binding region and is responsible for the interaction with PCNA [9]. The third region is the conserved zinc (Zn)-finger motif which assists DNA binding [9]. The C-terminal domain is specific to metazoan species and absent in yeast. The CTD assists the ID in binding to both DNA and the catalytic subunit of polα, and the combination of the two domains has higher affinity than either single domain alone [9]. There are two additional Zn-coordinating sites existing in the CTD distinct from that in the ID [16]. The first of these interacts with ssDNA, while second one shows homology to the Mcm2-7 helicase OB-fold Zn-ribbon [16].

Although extensive studies have been carried out on the role of Mcm10 in initiation of DNA replication, few studies have been reported that address the involvement of Mcm10 in the regulation of chromatin structure. Several studies in *S. cerevisiae* implicate Mcm10 in transcriptional repression of the mating type loci, and link DNA replication proteins to heterochromatin formation [17]–[19]. Analysis of synthetic gene arrays in *S. cerevisiae* showed that mutations of the *sin3* and *sds3* genes, encoding components of the Rpd3 complex, improved the viability of Mcm10 mutants [20]. Rpd3, a histone deacetylase is reported to be involved in suppressing late-origin firing through activation of the S-phase checkpoint [21], [22]. This finding implies that under conditions of Mcm10 depletion, it is beneficial to suppress histone deacetylation, possibly to allow for the activation of late-firing origins. Consistent with these observations, the depletion of Mcm10 in *Drosophila* cultured cells leads to under-condensed metaphase chromosomes [23]. This may point to a possible role for Mcm10 in chromatin structure and chromosome condensation. Additionally, analyses of a hypomorphic mutant of *Drosophila* Mcm10 (dMcm10) demonstrates that the protein has a role in heterochromatic silencing and chromosome condensation, while those with a C-terminal truncation allele of dMcm10 indicate that the CTD of dMcm10 is important for DNA replication [24]. These *in vivo* studies with *Drosophila* have been performed on the salivary glands and wing discs [24]. In the present study, we have characterized dMcm10 during compound eye development.

The *Drosophila* eye imaginal disc has been widely used for the study of DNA replication due to the highly synchronized mitotic waves which pass across the disc. In third instar larvae, the morphogenetic furrow (MF) appears at the posterior end of the eye imaginal discs and slowly moves in the anterior direction. Cells in front of the MF proliferate asynchronously, while those on the MF are arrested synchronously in the G1 phase. Cells behind the MF either leave the cell cycle and differentiate into the photoreceptors of the adult ommatidium, or undergo one more cell division. This cell cycle is a final synchronous round and produces an S-phase band (the second mitotic wave-SMW), after which these cells form a reservoir of cells for subsequent differentiation events. Mitotic cells are not normally present in the SMW [25].

*Drosophila* eye discs are also useful for development studies as defects in particular cell lineages can be easily observed. The *Drosophila* compound eye consists of nearly 800 ommatidia. Each ommatidial unit is surrounded by a hexagonal lattice of 12 interommatidial cells which are comprised of bristles, and secondary and tertiary pigment cells. It contains eight photoreceptor cells, four cone cells, and two primary pigment cells. The photoreceptors (R cells) are divided into three different types depending on their genetic and morphological function. The outer (R1–R6) lies in a ring surrounding two central receptors; R7, the distal, or outer, central cells; and R8, the proximal, or inner, central cells. R cells have been found to be generated sequentially: R8 is generated first, with movement posterior from the MF, then cells are added pair wise (R2 and R5, R3 and R4, and R1 and R6), and R7 is the last photoreceptor to be added to the precluster. Identification of each photoreceptor cell can be determined using enhancer trap lines expressing a nuclear-localized form of *E. coli* β-galactosidase controlled by the specific enhancer-promoter located nearby the P-element.

Here we analyze the role of *Drosophila* Mcm10 during eye development. A small and rough eye phenotype was observed in the *dMcm10* knockdown flies. We showed that the knockdown of *dMcm10* caused a delay in the S phase and subsequently lead to a delay in M phase. Furthermore, we found that knockdown of *dMcm10* also resulted in DNA damage and then induced apoptosis in cells. Surprisingly, a significant reduction of R7 photoreceptor cells in the eye imaginal discs was found in *dMcm10* knockdown flies crossed with enhancer trap lines. These defects in R7 cell differentiation appeared to be independent of apoptosis induction. Thus, *dMcm10* knockdown has an effect on both DNA replication and R7 cell differentiation.

## Materials and Methods

### Fly stocks

Fly stocks were reared at 25°C on standard food. Either yellow white flies or Canton S flies were used for the wild-type strain. Transgenic flies carrying UAS-*dMcm10IR_3-117_* were obtained from VDRC stock center carrying dsRNA that targets the region of dMCM10 from aa3 to aa117 [26]. Enhancer trap lines carrying the lacZ markers B38 (inserted in *klingon*), P82 (inserted into *deadpan*) were obtained from Dr. Y. Hiromi. These lines express the β-galactosidase marker in photoreceptor cells (R) of R7 or R3/R4/R7. All other stocks used in this study were obtained from Bloomington *Drosophila* stock center or Kyoto *Drosophila* Genetic Resource Center.

### Establishment of transgenic flies

The dMcm10 cDNA fragment encoding amino acids (aa) 633-700 was obtained by RT-PCR with total RNA isolated from *Drosophila* whole bodies of adult flies. The fragment was cloned into the pWIZ vector [27] in normal and reversed orientations to create pUAS-*dMcm10IR_633-700_*. The full length dMcm10 cDNA fragment was amplified with the primers 5′-*EcoR*I-HA-dMcm10 (5′-CGAATTCATGGCTTACCCATACGATGTTCCAGATTACGCTATGGGTCCTGCTGAGAAATC) and 3′-*Xho*I-dMcm10-2 (5′-CAACTCGAGTCACTCTTGATCGGGTACCA) and inserted into *EcoR*I and *Xho*I sites of pUAST [28] to create pUAS-*HA-dMcm10*. P element-mediated germ line transformation was carried out as described earlier [29] and F1 transformants were selected on the basis of white-eye color rescue [30]. Two independent lines were established for pUAS-*dMcm10IR*. These lines in which the transgene was inserted in the second and third chromosome, respectively showed essentially same phenotype. In the knockdown studies we mainly used the line carrying the UAS-*dMcm10IR_633-700_* gene on the third chromosome. Only one line was established for the over-expression line carrying UAS-*HA-dMcm10* gene on the X chromosome.

### Preparation of anti-dMcm10 antibody

To generate pGEX-dMcm10, a cDNA fragment was amplified by RT-PCR and the PCR product was inserted into pGEX-6T-1 (GE healthcare). The recombinant GST-dMcm10 protein was expressed in *E. coli* BL21. Lysates of cells were prepared by sonication in PBS containing 1 mM PMSF, and 1 μM each of pepstatin and leupeptin. The recombinant protein was purified with a glutathione-Sepharose column (GE healthcare). Then, the purified Mcm10 protein was used to elicit polyclonal antibody production in rabbit. Polyclonal antibodies reacting with dMcm10 were affinity-purified from anti-serum using the NHS-activated Sepharose HP (GE healthcare) coupled with GST-dMcm10 fusion protein after passing through the GST-conjugated Sepharose HP.

### Western immunoblot analysis

Third instar larvae of flies with the following genotypes: Canton S; *yw*, +, Act5C-GAL4/UAS-*HA-dMcm10*; and *yw*, +, Act5C-GAL4/UAS-*dMcm10IR_633-700_* were washed in PBS and homogenized in an extraction buffer containing 50 mM Tris-HCl (pH7.5), 5 mM MgCl_2_, 150 mM NaCl, 10% glycerol, 0.1% Triton X-100, 0.1% NP-40, 10 μg/ml each of aprotinin, leupeptin, pepstatin A and 1 g/ml each of antipain, chymostatin and phosphoramidon. Homogenates were centrifuged, and 20 μg of protein from each of these extracts was electrophoretically separated on 10% polyacrylamide gels containing 10% SDS and transferred to Polyvinilidenedifluoride (PVDF) membrane (BIO-RAD) in a solution containing 25 mM Tris-HCl, 192 mM glycine and 20% methanol at 25°C for 2 h. The blotted membranes were blocked with a blocking buffer containing 20 mM Tris-HCl (pH 7.4), 150 mM NaCl and 5% skimmed milk at 25°C for 1 h and then incubated with a rabbit anti-dMcm10 antibody at 1∶5000 dilution or mouse anti-α-tubulin monoclonal antibody (Sigma-Aldrich) at 1∶8000 dilution at 4°C for 16 h. After washing with TBS containing 0.05% Tween20, the blots were incubated with horseradish peroxidase-labeled rabbit anti-IgG (GE healthcare) at 1∶20,000 dilution or mouse anti-IgG (GE healthcare) at 1∶10,000 dilution at 25°C for 1 h. Detection was performed with ECL Western blotting analysis system (GE healthcare) with LumivisionPro HS II image analyzer (Aisin seiki).

### Scanning electron microscopy

Adult flies were anesthetized, mounted on stages and observed with a VE-7800 (Keyence Inc.) scanning electron microscope or JSM-6510A (JEOL) analytical scanning electron microscope. In every experiment, at least five adult flies of each line were chosen for scanning electron microscopy observation to assess the eye phenotype, and these experiments were repeated 3 times. In the experiments, no significant variation in eye phenotype among the five individuals was observed.

### Immunostaining

For immunostaining, third instar larval eye imaginal discs were dissected and fixed in 4% paraformaldehyde in PBS for 15 min at 25°C. After washing with PBS containing 0.3% Triton X-100 (PBST), samples were blocked with PBS containing 0.15% Triton X-100 and 10% normal goat serum for 30 min at 25°C and incubated with diluted primary antibodies in PBS or in PBS containing 0.15% Triton X-100 and 10% normal goat serum for 16 h at 4°C. The following antibodies were used as primary antibodies: rabbit anti-dMcm10 (diluted at 1∶100), mouse anti-*Drosophila* polymerase α (dpolα) (1∶200, [31]), rabbit anti-cleaved Caspase-3 (1∶100, BD Biosciences), mouse anti-β-galactosidase antibody (1∶400, DSHB), rabbit anti-Ser10 phosphorylated histone H3 (PH3, 1∶200, Cell Signaling Technology), rabbit anti-phospho H2AvD (1∶200, Rockland), mouse anti-Prospero (1∶400, DSHB). After extensive washing with PBST, samples were incubated with secondary antibodies labeled with either Alexa594 or Alexa488 (1∶400; Invitrogen) for 3 h at 25°C. After further washing with PBST and PBS, samples were mounted in Vectashield Mounting Medium (Vector laboratories) and inspected with a confocal laser scanning microscope (Olympus FLUOVIEW FV10i).

### 5-ethynyl-2′-deoxyuridine (EdU) labeling

Detection of cells in S phase was performed using an EdU-labeling kit from Invitrogen (Click-iT EdU Alexa Fluor 594 Imaging Kit). Third instar larvae were dissected in PBS and the imaginal discs were suspended in Grace's insect medium in the presence of 10 μM EdU for 60 min at 25°C. The samples then were fixed with 3.7% Formaldehyde in PBS for 15 min at 25°C. After fixing, samples were washed with 3% BSA in PBS and were permeabilized in 0.5% Triton X-100 in PBS for 20 min at 25°C. Then, samples were washed with 3% BSA in PBS and incubated with Click-iT reaction cocktails for 30 min at 25°C (following the manufacturers' instructions). After further washing with 3% BSA in PBS and PBS, samples were stained with Hoechst 33342 (Invitrogen) for labeling DNA, and finally samples were mounted and observed as described in immunostaining section.

### Flip-out experiments

RNAi clones in eye discs were generated with a flip-out system [32]. Female flies with *hs-flp*; *Act5*C>FRT y FRT>GAL4, UAS-*GFP* were crossed with UAS-*dMcm10IR* and clones were identified by the presence of green fluorescent protein (GFP) expressed under control of the *Act*5C promoter. Flip-out was induced by heat shock (60 min at 37°C) 24–48 h after the eggs were laid.

### Quantification and Statistical analysis

The phospho-H2AvD, phospho-Histone H3 positive cells and EdU signals in the region posterior to the MF were counted and measured from six eye imaginal discs by using Meta Morph software. The experiments were repeated three times. Then, statistical analysis was conducted, as indicated in the figure legends, using GraphPad Prism 6. Every single set of data was calculated by using Welch's t-test. Significance is as described in the figure legends with *p<0.05; **p<0.01; ***p<0.001; ****p<0.0001.

## Results

### Localization of dMcm10 in the nucleus of salivary glands cells

To confirm the specificity of Mcm10 antibody, we conducted Western immunoblot analyses. A single 86 kDa band was detected with protein extracts from third instar larvae of Canton S (Figure S1, lane 2). The size is consistent with the predicted size of the dMcm10 protein from the amino acid composition (86,524 Da). The level of this 86 kDa band was increased in third instar larval extracts of *Act5C*-GAL4>UAS-*HA-dMcm10* flies (Figure S1, lane 1). Moreover, the 86 kDa was significantly reduced in extracts from *Act5C*-GAL4>UAS-*dMcm10IR_633-700_* flies (Figure S1, lane 3). These results indicate that the anti-dMcm10 antibody can specifically detect dMcm10 protein, and also confirmed that the RNAi line shows an efficient knockdown of *dMcm10*.

**Figure 1:**
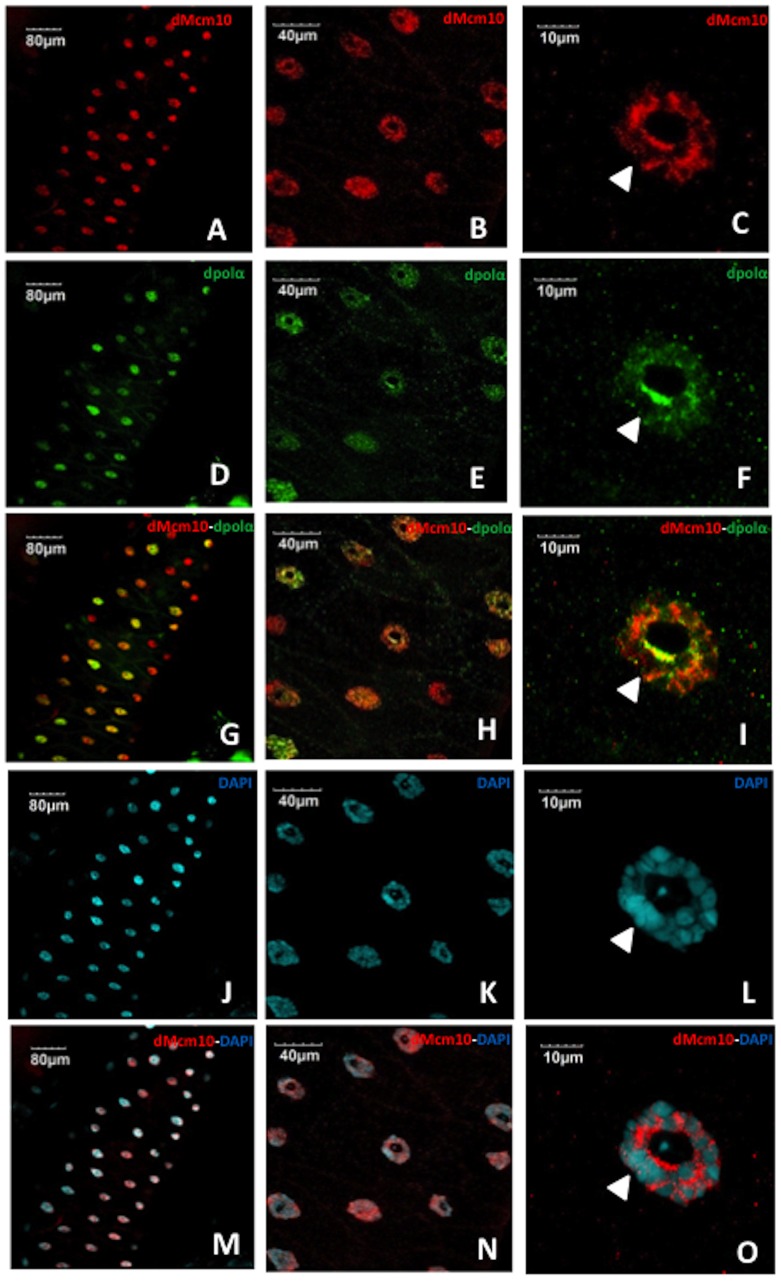
Mcm10 localizes mainly in the nucleus of salivary gland cells. The salivary glands of third instar lavae of Canton S were stained with anti-dMcm10 antibody (Red) (A, B, C), with anti-dpolα antibody (Green) (D, E, F) and with DAPI for DNA staining (Blue) (J, K, L). Merged images for anti-dMcm10 antibody and anti-dpolα antibody (G, H, I), and anti-dMcm10 and DAPI (M, N, O) are also shown. B, E, H, K, N and C, F, I, L, O show sequentially enlarged images of A, D, G, J, and M, respectively. Bars indicate 80 μm, 40 μm, 10 μm respectively. White arrowheads indicate the heterochromatic chromocenter.

Salivary gland cells in *Drosophila* proliferate by repeated rounds of endoreplication, consisting of only S- and G-phase, to form a giant polytene chromosome. Thus, salivary gland cells provide a unique model to visualize the specific chromatin localization patterns of individual proteins. Immunostaining of the salivary glands from Canton S flies with anti-dMcm10 antibody showed that dMcm10 mainly localized in the nucleus of the cells (Figure 1A). Immunostaining with anti-dpolα antibody also showed that polα mainly localized in the nucleus (Figure 1D). Although dMcm10 signals were evenly detected in most nuclei in the salivary gland, dpolα signals were higher in some nuclei than others (Figure 1A, D and G). In higher magnification images of the nucleus, both dMcm10 signals and dpolα signals appear to be excluded from the nucleolus and DAPI-dense heterochromatic chromocenter (Figure 1C, F, I, L and O). These results suggest that both proteins mainly localize on euchromatic regions of the polytene chromosome in nuclei, although clarification of this point will require more detailed examination of polytene chromosome preparations. In addition, double staining with anti-dMcm10 antibody and anti-dpolα antibody showed that both dMcm10 and dpolα only partially co-localize in polytene nuclei (Figure 1C, F and I). The observed co-localization is consistent with previous studies indicating that Mcm10 binding to the catalytic subunit of polα is required for chromatin association [2], [7], [14]–[16], [33].

### Knockdown of *dMcm10* in eye imaginal discs induces morphologically aberrant eyes in adult flies

To examine the role of dMcm10 during *Drosophila* development, we established UAS*-dMcm10IR_633-700_* transgenic fly strains that incorporated dsRNA targeting the region between aa633 and aa700. The UAS-*dMcm10IR_633-700_* line was then crossed to several GAL4 driver lines (Table S1). Expression of double stranded RNA (dsRNA) that targeted dMcm10 in the whole body using the *Act5C*-GAL4 driver caused pupal lethality, indicating that dMcm10 is essential for viability.

**Figure 2:**
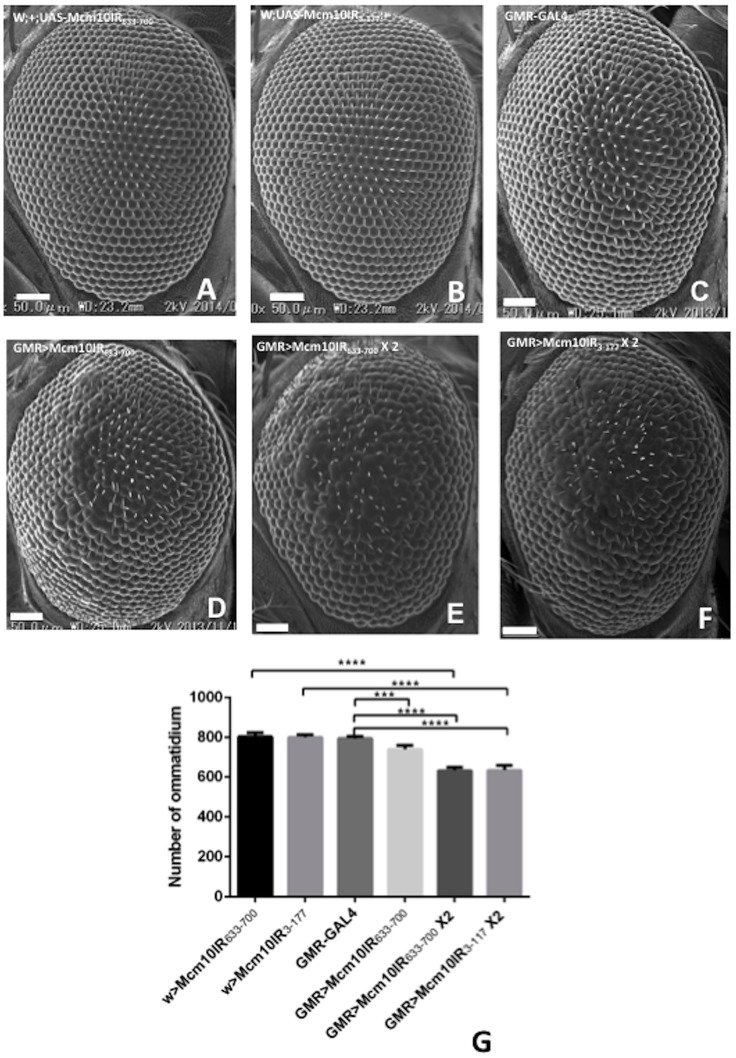
Knockdown of *dMcm10* in eye imaginal discs causes a small and rough eye phenotype. Scanning electron micrographs of adult compound eyes. Posterior is to the right and dorsal is to the top. The bar indicates 50 μm. (A) *w*; +; UAS-*Mcm10IR_633-700_*; (B) *w*; UAS-*Mcm10IR_3-117_*; +; (C) *GMR*-GAL4/+; +; +; (D) *GMR*-GAL4/+; +; UAS-*dMcm10IR_633-700_*/+; (E) *GMR*-GAL4/+; +; UAS-*Mcm10IR_633-700_*/UAS-*MCM10IR_633-700_*; (F)*GMR*-GAL4/+;UAS-*Mcm10IR_3-117_*/UAS-*Mcm10IR_3-117_*;+; (G) Quantification of the number of ommatidium in adult eye flies. Mean intensities with standard deviation from six adult eyes are shown. ***p<0.001, ****p<0.0001. The flies were reared at 28°C.

Two dMcm10 RNAi lines lacking the *GMR*-GAL4 driver and the line with *GMR*-GAL4 alone show normal eye phenotypes (Figure 2A, B and C). Specific expression of dsRNA in eye imaginal discs by the *GMR*-GAL4 driver resulted in a morphologically aberrant rough eye phenotype. The fly line with 1 copy of *dMcm10* dsRNA (Figure 2D) is less rough than the fly line with 2 copies of *dMcm10* dsRNA (Figure 2E). In addition, two different *dMcm10* knockdown flies including *dMcm10IR_633-700_* and *dMcm10IR_3-117_* showed a similar extent of rough eye phenotype and a similar reduction in the number of ommatidium (Figure 2E and F) in comparison with control flies (Figure 2A, B, and C) resulting in small eye phenotype. This excludes the possibility that the phenotypes that we are observe are due to off-target effects.

**Figure 3:**
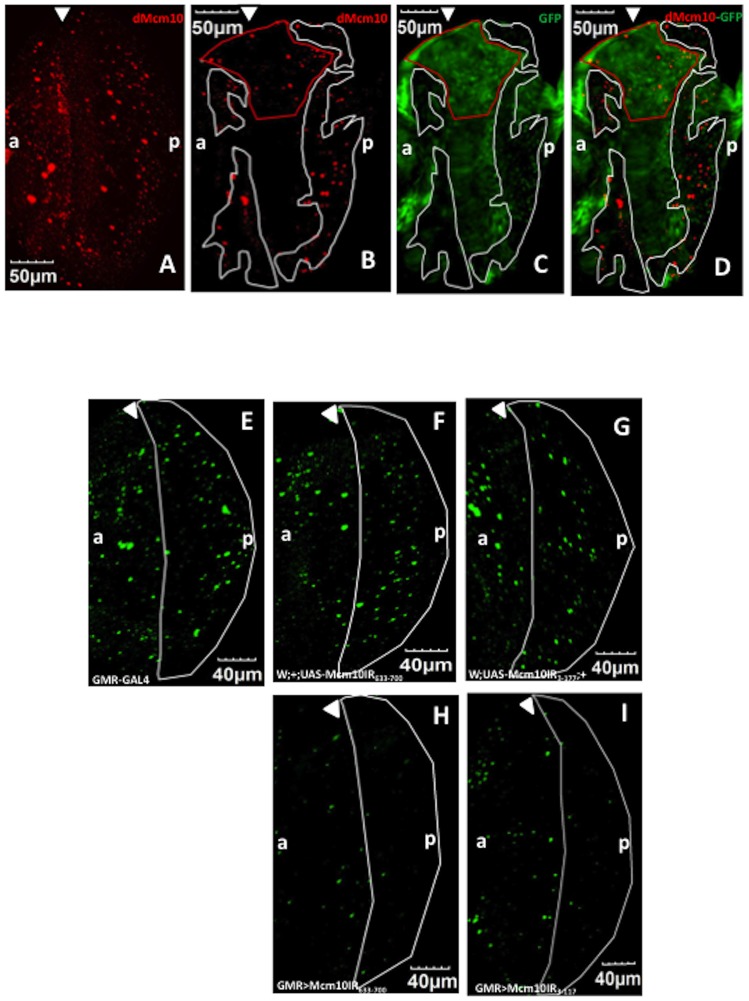
Expression of *dMcm10* dsRNA reduces dMcm10 levels in eye discs. In the flip out experiment: (A) Eye imaginal discs of Canton S are stained with anti-dMcm10 antibody (Red). (B) Eye discs expressing *dMcm10* dsRNA are stained with anti-dMcm10 antibody (Red). (C) Cells expressing *dMcm10* dsRNA are marked with GFP (Green). (D) Merged image of anti-dMcm10 and GFP signals in *dMcm10* knockdown eye discs. The white border lines show the GFP-negative region in which *dMcm10* dsRNA is not expressed. The red border line shows the region in which the eye disc is folded back and some apparently overlapped signals in this region. In the experiment for *dMcm10* knockdown by *GMR*-GAL4 driver: (E) *GMR*-GAL4: +: +; (F) *w*; +; UAS-*Mcm10IR_633-700_*, (G) *w*; UAS-*Mcm10IR_3-117_*; +; (H) *GMR*-GAL4; +; UAS-*Mcm10IR_633-700_*; (I) *GMR*-GAL4: UAS*-Mcm10IR_3-117_*; +. (E-I) eye imaginal discs are stained with anti-dMcm10 antibody (Green). (E–G) dMcm10 signals are observed everywhere in the eye discs including the posterior regions (white border line). However, in two independent knockdown flies (H and I), there is a significant reduction of dMcm10 signals in the posterior regions. White arrowheads indicate morphogenetic furrow (MF). The bars indicate 50 μm and 40 μm respectively. (a) indicates anterior, (p) indicates posterior.

The targeted effects of *dMcm10* dsRNA on dMcm10 expression in the eye disc were confirmed by a flip-out experiment. Immunostaining of eye imaginal discs of wild type flies with anti-dMcm10 antibody revealed that dMcm10 is expressed ubiquitously in eye discs, with slightly higher expression in the S-phase zone behind the morphogenetic furrow (Figure 3A). In the flip-out experiments, cells marked with GFP (Green) expressed *dMcm10* dsRNA (Figure 3C). The GFP marked cells showed decreased levels of dMcm10 signals (Red) (Figure 3B and D) confirming the specific knockdown of *dMcm10* in the eye disc by expression of *dMcm10* dsRNA.

In order to further confirm the efficiency of RNAi, we performed additional immunostaining on control and knockdown flies with anti-dMcm10 antibody (Green). Many dMcm10 signals appear as dots in the posterior region of the eye discs in *GMR*-GAL4 alone and two independent dMcm10 RNAi lines without *GMR*-GAL4 driver (Figure 3E, F and G). However, there was a significant decrease in the density of dMcm10 signals in those posterior regions expressing *dMcm10* dsRNA in the *dMcm10* knockdown flies (Figure 3H and 3I). Furthermore, the same patterns were observed in two independent *dMcm10* knockdown flies targeting different regions of Mcm10. These results further confirm the specific knockdown of *dMcm10* in the eye disc by expression of *dMcm10* dsRNA, and also exclude possible off-target effects.

### Knockdown of *dMcm10* induces delays in S phase and M phase in the eye disc cells

**Figure 4:**
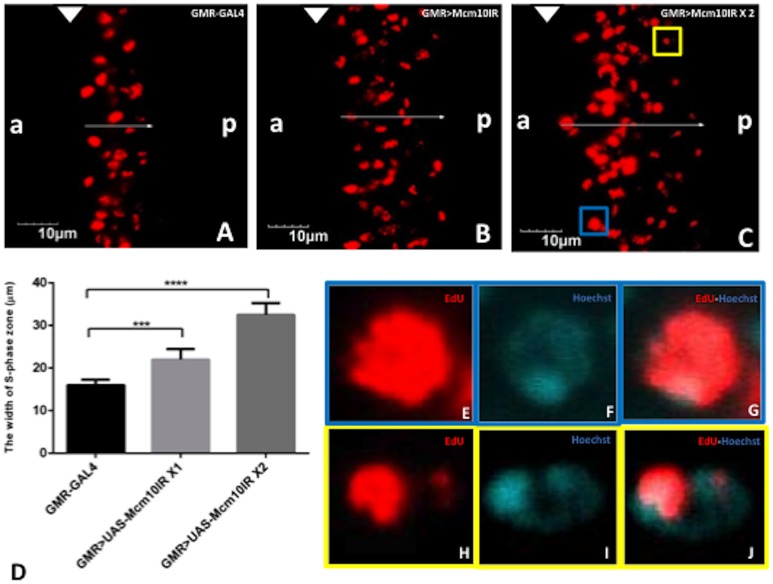
Knockdown of *dMcm10* in eye imaginal discs induces a delay in S phase. The eye imaginal discs were labeled with EdU (Red) (A) *GMR*-GAL4/+; +; +; (B) *GMR*-GAL4/+; +; UAS-*Mcm10IR_633-700_*/+; (C) *GMR*-GAL4/+; +; UAS-*Mcm10IR_633-700_*/UAS-*Mcm10IR_633-700_*. The arrows show the positions measured. (D) Quantification of the width of S phase zone in the posterior region. ***p<0.001, ***p<0.0001. The yellow square shows the position of an EdU cell possibly undergoing replication of the late replicating heterochromatic region. The blue square shows the position of an EdU cell possibly undergoing replication of the early replicating euchromatic region. Figure E–G present higher magnification images of the blue square. Figure H–J present higher magnification images of the yellow square. Cells were stained with EdU (Red) (E and H) or Hoechst 33342 (Blue) (F and I). Figures G and J are merged images of both EdU and Hoechst 33342. The bars indicate 10 mm. The flies were reared at 28°C. White arrowheads indicate morphogenetic furrow (MF). (a) indicates anterior, (p) indicates posterior.

The effect of *dMcm10* knockdown on the cell cycle progression in eye imaginal discs was examined. An EdU incorporation assay was used to monitor S phase, and immunostaining with the anti-phospho-histone H3 (PH3) antibody to monitor M phase cells. In third instar larvae, the morphogenetic furrow moves from posterior to anterior in eye discs. Cells in the region anterior to the morphogenetic furrow proliferate at random. All cells in the morphogenetic furrow are arrested in G1 phase and then they undergo one round of cell cycle consisting of G1, S, G2, M phases in a highly synchronized fashion before entering G0. The *GMR-*GAL4 driver expresses GAL4 in cells from morphogenetic furrow to the posterior end in eye discs. The EdU incorporation assay detected the synchronized S phase cells behind the morphogenetic furrow (MF) in the eye discs (Figure 4). EdU positive cells towards the anterior region are bigger and EdU signals coincide fully with Hoechst signals, representing early replicating euchromatic regions (Figure 4E–G). In contrast, EdU spots become smaller in the posterior side of the S phase zone and the signal coincides with the Hoechst bright spot in each nucleus, likely representing late replicating heterochromatic regions (Figure 4H–J). The zone of synchronized S phase in eye imaginal discs was measured in order to evaluate whether there is an S phase delay in the knockdown flies. The wider zone of S phase cells detected in *dMcm10* knockdown flies indicated that more cells were delayed in S phase progression (Figure 4B and C). The quantified data indicate an increase of 1.3 fold for flies carrying one copy of UAS-*dMcm10IR_633-700_*, and an increase of 1.9 fold for those carrying two copies of UAS-*dMcm10IR_633-700_*, compared to the flies carrying *GMR*-GAL4 alone (Figure 4D). Similar results were also obtained for a different *dMcm10* knockdown fly line, carrying UAS-*dMcm10IR_3-117_* (Figure S2).

**Figure 5:**
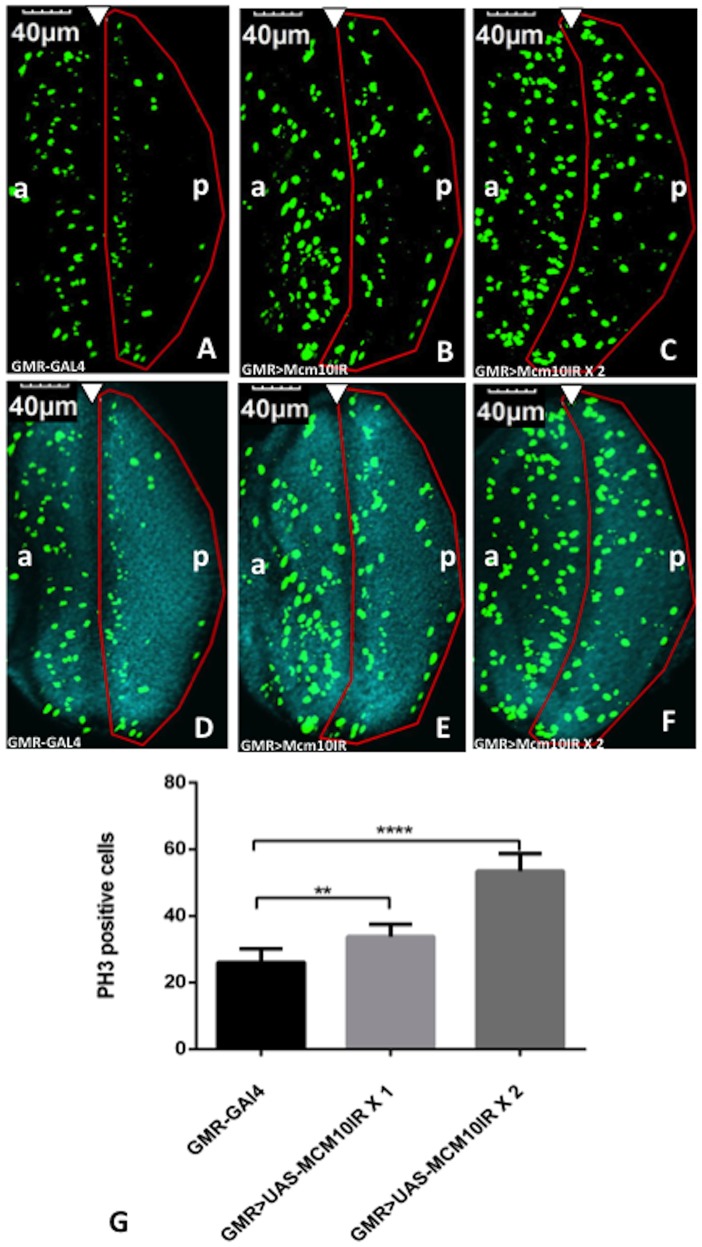
Knockdown of *dMcm10* in eye imaginal discs induces a delay in M phase. The eye imaginal discs were stained with anti-Ser10 phosphorylated histone H3 (PH3) antibody (Green) (A, B, C). Also shown are merged images of the eye discs stained with DAPI for DNA (Blue) and anti PH3 antibody (Green) (D, E, F). (A, D) *GMR*-GAL4/+; +; +; (B, E) *GMR*-GAL4/+; +; UAS-*Mcm10IR_633-700_*/+; (C, F) *GMR*-GAL4/+; +; UAS-*Mcm10IR_633-700_*/UAS-*Mcm10IR_633-700_*. The white arrowhead shows the morphogenetic furrow. The flies were reared at 28°C. Bars indicate 40 μm. (G) Quantification of phosphorylated histone H3 positive index in the posterior region of the eye discs. Flies carrying two copies of UAS-*dMcm10IR_633-700_* caused a significant increase in the number of mitotic cells in the posterior region compared to control flies carrying *GMR*-GAL4 alone. Flies carrying one copy of UAS-*dMcm10IR_633-700_* also caused a delay in M phase, but not as much as with two copies. **p<0.01, ****p<0.0001. The red border line indicates the posterior region. (a) indicates anterior, (p) indicates posterior.

A delay in S phase may subsequently lead to a delay in the M phase. Therefore, we carried out immunostaining of the third instar larval eye discs with anti-PH3 antibody, to examine the distribution of M phase cells in the posterior regions. In the eye discs of control flies, the mitotic cells formed a vertical line in the synchronized M phase zone and only a few scattered cells were observed in the posterior region (Figure 5A). However, in the *dMcm10* knockdown flies, the vertical line of mitotic cells was diffused into the posterior region (Figure 5B). A further increase of the dose of UAS-*dMcm10IR_633-700_* resulted in more diffusion of mitotic cells into the posterior area (Figure 5C). We also detected an increase in the total number of mitotic cells in the region posterior to the morphogenetic furrow of eye discs in the *dMcm10* knockdown flies (Figure 5B and 5C) in comparison with control flies (Figure 5A). Quantitative analyses revealed a 1.3 fold increase for the flies carrying one copy of UAS-*dMcm10IR_633-700_*, and a 2.1 fold increase for those carrying two copies of *dMcm10IR_633-700_,* compared to the flies carrying *GMR*-GAL4 alone (Figure 5G). These results indicate that knockdown of *dMcm10* not only caused delayed entry in M phase but also resulted in delayed progression of M phase.

### Knockdown of *dMcm10* induces DNA damage and apoptosis in eye imaginal discs

**Figure 6:**
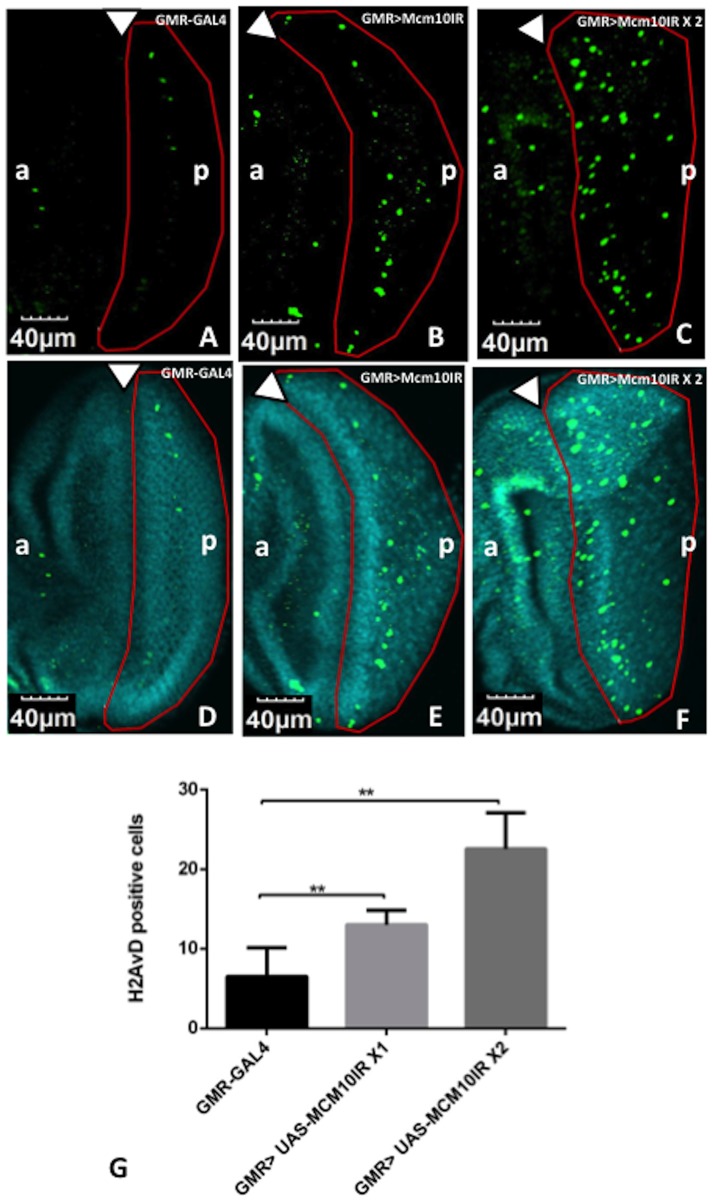
Knockdown of *dMcm10* in eye imaginal discs induces DNA damage. The eye imaginal discs were stained with anti-phospho H2AvD antibody (Green) (A, B, C). Also shown are merged images of the eye discs stained with DAPI for DNA (Blue) and anti-phospho H2AvD antibody (Green) (D, E, F). (A, D) *GMR*-GAL4/*yw*; +; +; (B, E) *GMR*-GAL4/*yw*; +; UAS-*Mcm10IR_633-700_*/+; (C, F) *GMR*-GAL4/*yw*; +; UAS-*Mcm10IR_633-700_*/UAS-*Mcm10IR_633-700_*. The white arrowhead shows the morphogenetic furrow. The flies were reared at 28°C. Bars indicate 40 μm. (G) Quantification of phospho-H2AvD positive index in the posterior region of eye discs. There was an increase in the phospho-H2AvD positive signals with two copies of *dMcm10* dsRNA in the posterior region compared to control, *GMR*-GAL4 alone. One copy of the dsRNA also caused an increase in DNA damage signal, but not as much as for two copies. **p<0.01. The red border line indicates the posterior region. (a) indicates anterior, (p) indicates posterior.

The S phase block and delay in both entry and progression of M phase could cause defects in the stability of forks or condensed chromosomes. Therefore, we examined whether the knockdown of *dMcm10* could cause genomic DNA damage. The eye discs of *dMcm10* knockdown flies were immunostained with DAPI to visualize the DNA and antibodies against the phospho-H2AvD protein (a *Drosophila* homologue of H2Ax) to visualize DNA damage sites in the chromatin. The immunostaining data showed that more phospho-H2AvD positive cells were detected in the posterior region of *dMcm10* knockdown eye discs (Figure 6B and C) than in control flies (Figure 6A). This is also confirmed by quantitative analyses of the number of H2AvD positive cells in the posterior region (Figure 6G).These data indicate that dMcm10 depletion causes DNA damage.

**Figure 7:**
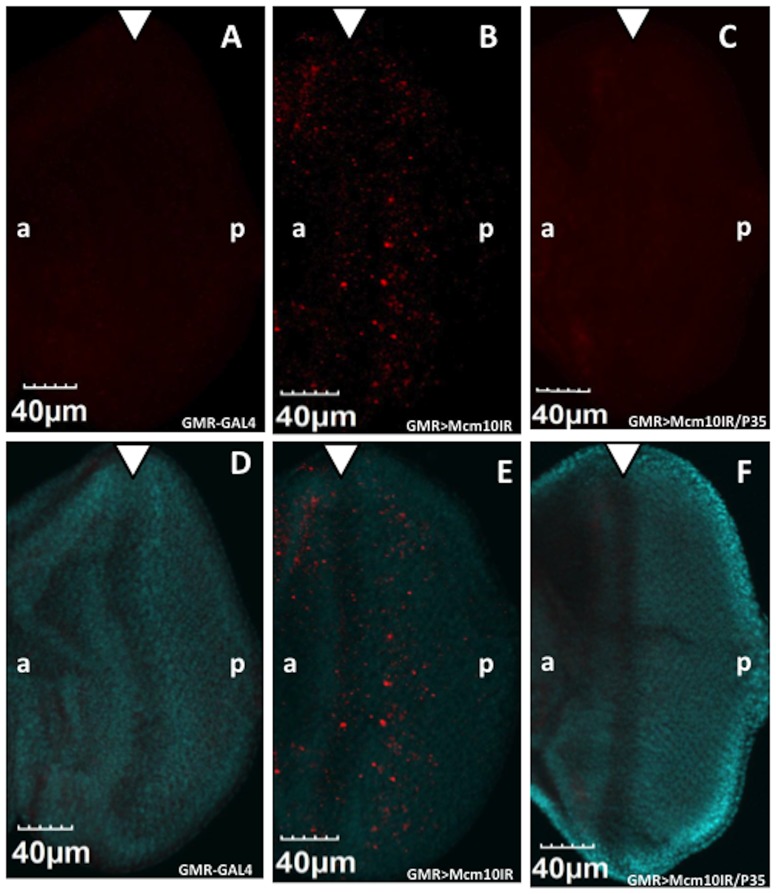
Knockdown of *dMcm10* in eye imaginal discs induces apoptosis. The eye imaginal discs of third instar lavae were stained with anti-cleaved caspase-3 antibody(A, B, C) (Red). The merged images of eye discs stained with DAPI for DNA (Blue) and anti-cleaved caspase-3 antibody (Red) (D, E, F). (A, D) *GMR*-GAL4/*yw*; +; +; (B, E) *GMR*-GAL4/*yw*; +; UAS-*Mcm10IR_633-700_*/+; (C, F) *GMR*-GAL4/*yw*; +; UAS-*Mcm10IR_633-700_*/UAS-*P35*. White arrowhead shows morphogenetic furrow. The flies were reared at 28°C. Bars indicate 40 μm.

**Figure 8:**
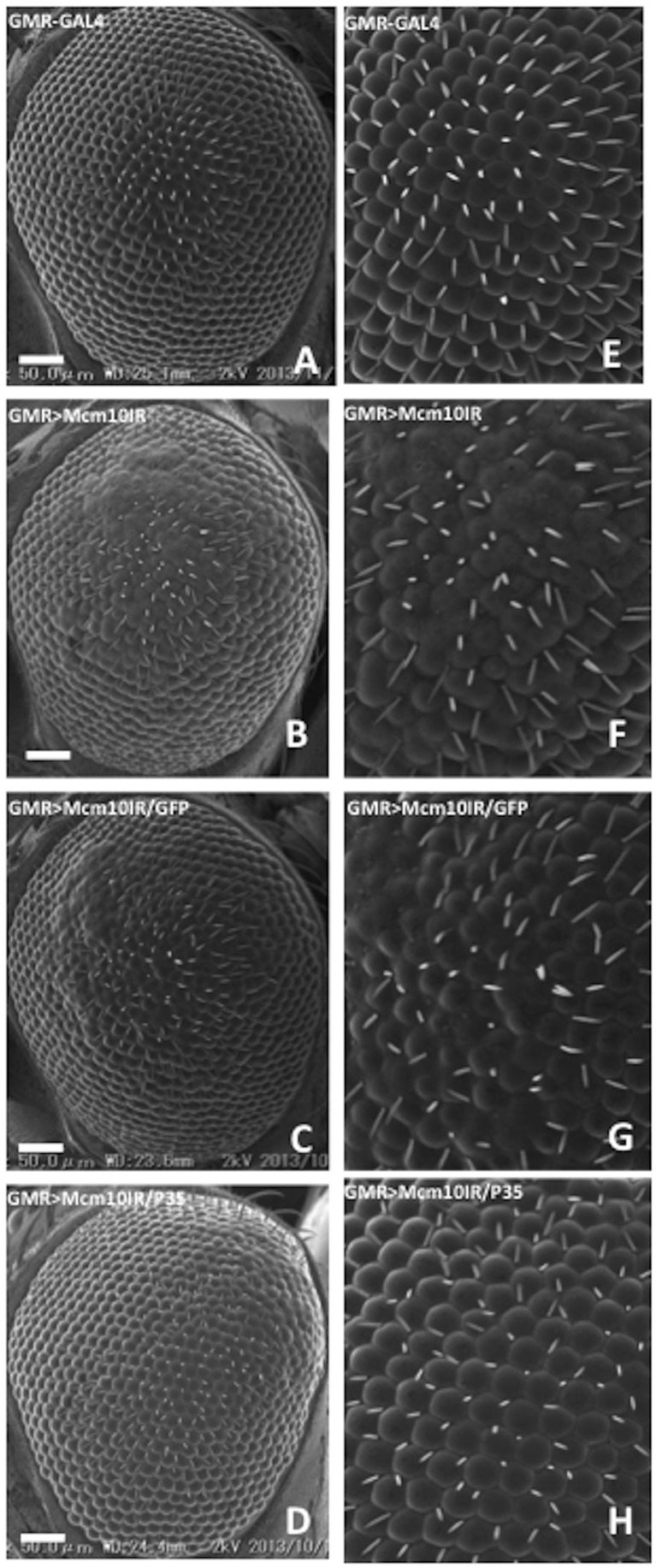
Expression of apoptosis inhibitor P35 suppressed the rough eye induced by knockdown of *dMcm10*. Scanning electron micrographs of adult compound eyes. Posterior is to the right and dorsal is to the top. The flies were reared at 28°C. The bar indicates 50 μm. (A) *GMR*-GAL4/*yw*; +; +; (B) *GMR*-GAL4/*yw*; +; UAS-*Mcm10IR_633-700_*/+; (C) *GMR*-GAL4/*yw*; +; UAS-*Mcm10IR_633-700_*/UAS-*GFP*; (D) *GMR*-GAL4/*yw*; +; UAS-*Mcm10IR_633-700_*/UAS-*P35*. E, F, G, and H show enlarged images of A, B, C, and D, respectively. White bars indicate 50 μm. The flies were reared at 28°C.

To determine whether DNA damage in cells can lead to an induction of apoptosis, immunostaining was carried out using antibodies against the anti-cleaved Caspase-3, a key mediator of apoptosis. In eye imaginal discs of flies expressing GAL4 alone, very few apoptotic cells were detectable. However, eye imaginal discs of *dMcm10* knockdown flies showed a significant increase in cell death signals in the region posterior to the morphogenetic furrow (Figure 7B and E) compared to the control (Figure 7A and D). Apoptosis is often associated with a rough eye phenotype. Therefore in order to confirm that the apoptosis is specifically induced by knockdown of *dMcm10* in eye imaginal discs, *GMR*-GAL4>UAS-*Mcm10IR_633-700_* flies were crossed with flies expressing an apoptosis inhibitor, P35. An obvious partial rescue of the rough eye phenotype induced by knockdown of *dMcm10* was found in flies co-expressing P35 (Figure 8D and H). Co-expression of P35 also suppressed the ectopic induction of apoptosis signals (Figure 7C and F). However, the rough eye phenotype was not suppressed in *dMcm10* knockdown flies co-expressing GFP (Figure 8C and G). Taken together, these observations provide further evidence for an important role for dMcm10 in genomic stability.

### Knockdown of *dMcm10* affects the differentiation of the R7 photoreceptor cell

Immunostaining of eye discs with anti-dMcm10 showed several enhanced dMcm10 signals accumulating in the posterior regions where photoreceptor cell differentiation occurs (Figure 3A). These observations lead us to examine whether dMcm10 plays a role in the differentiation of photoreceptor cells. Photoreceptor cells have been found to be generated sequentially: R8 is generated first, with movement posterior from the MF, then cells are added pair wise (R2, and R5, R3 and R4, and R1 and R6), R7 being the last photoreceptor to be added to the precluster. Several enhancer trap lines express a nuclear-localized form of *E. coli* β-galactosidase depending on the specific enhancer-promoter located near the P-element insertion.

**Figure 9:**
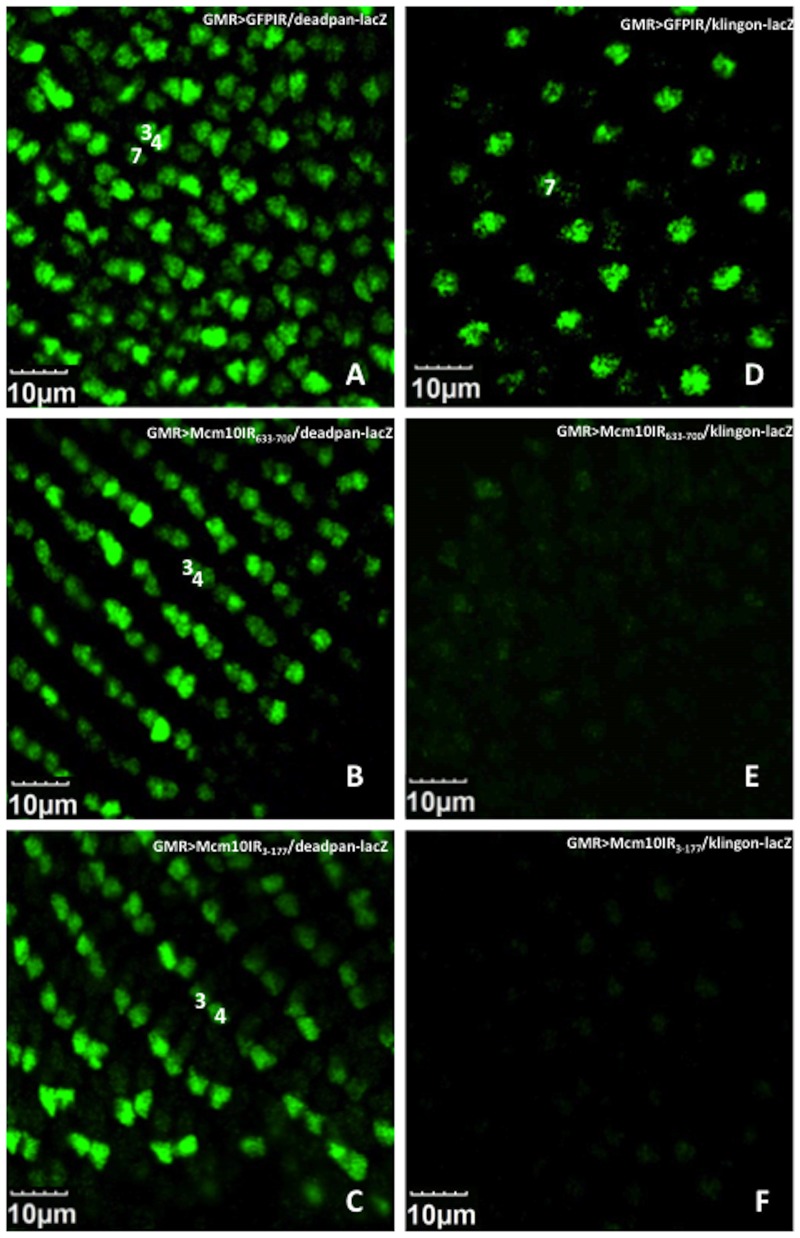
Effect of *dMcm10* knockdown on photoreceptor cells monitored by two enhancer trap lines. Immunostaining of eye discs with anti-lacZ antibodies (Green) (A) *GMR*-GAL4/+; deadpan-lacZ/+; UAS-*GFPIR*/+; (B)*GMR*-GAL4/+; deadpan-lacZ/+; UAS-d*Mcm10IR_633-700_*/+; (C) *GMR*-GAL4/+; deadpan-lacZ/UAS-d*Mcm10IR_3-117_*, +; (D) *GMR*-GAL4/+; +; UAS-*GFPIR*/klingon-lacZ, (E) *GMR*-GAL4/+; +; UAS-*Mcm10IR_633-700_*/klingon-lacZ; (F) *GMR*-GAL4/+; UAS-d*Mcm10IR_3-117_*/+; klingon-lacZ/+. (A–C) R3, R4, and R7 photoreceptor cells are marked by P82 (deadpan-lacZ) and (D–F) R7 photoreceptor cells are marked by B38 (klingon-lacZ). R7 photoreceptor cells were reduced significantly in both of the knockdown lines: *dMcm10IR_633-700_* (B, E) and *dMcm10IR_3-117_* (C, F). Bars indicate 10 μm. The flies were reared at 28°C.

**Figure 10:**
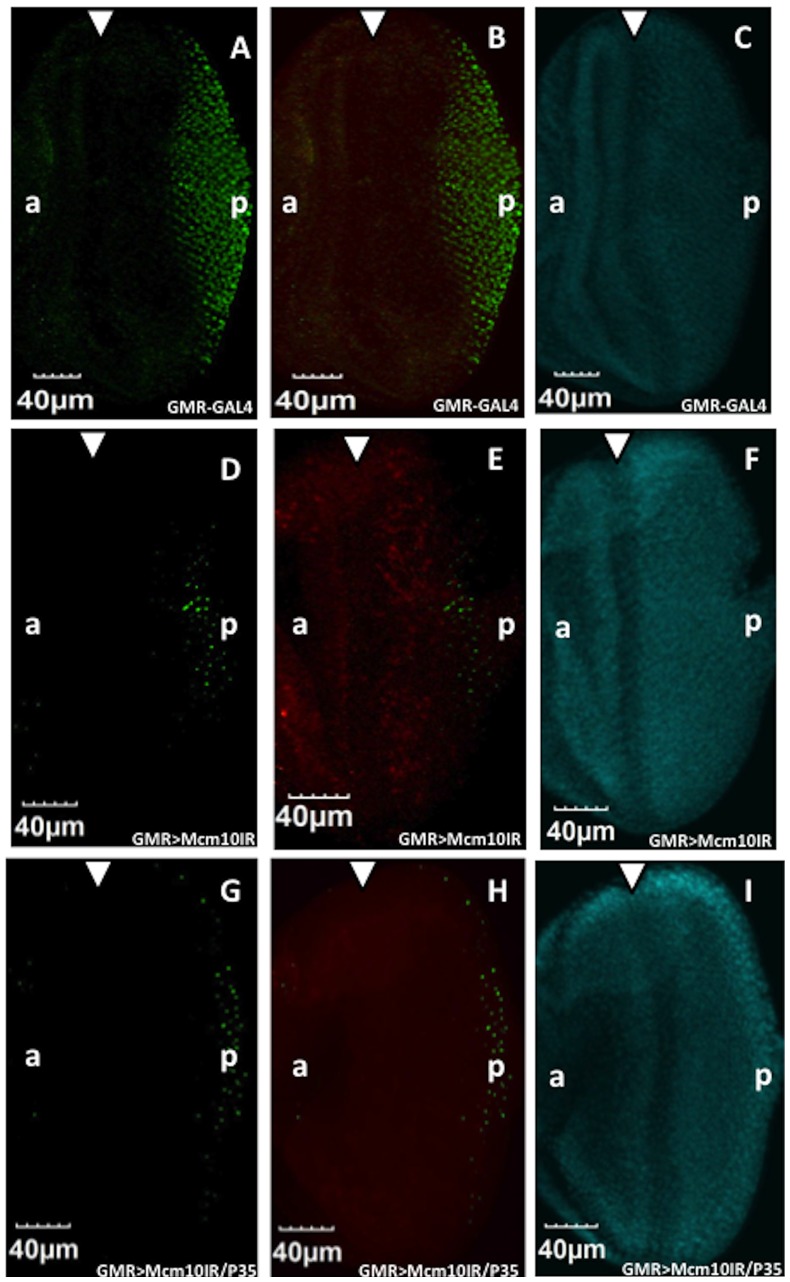
Knockdown of *dMcm10* in eye imaginal discs inhibits R7 photoreceptor cell differentiation independently of its apoptotic effect. The eye imaginal discs of third instar lavae were stained with anti-Prospero antibody (Green) (A, D, G). Merged images were generated of eye discs staining with anti-Prospero antibody (Green) and anti-cleaved caspase-3 antibody (Red) (B, E, H). Images of eye discs staining DNA with DAPI (Blue) (C, F, I) are also shown. (A, B, C) shows *GMR*-GAL4/*yw*; +; +; (D, E, F) shows *GMR*-GAL4/*yw*; +; UAS-d*Mcm10IR_633-700_*/+; and (G, H, J) shows *GMR*-GAL4/*yw*; +; UAS-d*Mcm10IR_633-700_*/UAS-*P35*. White arrowhead shows morphogenetic furrow. Bars indicate 40 μm. The flies were reared at 28°C.

To examine whether knockdown of *dMcm10* induces abnormal development of photoreceptors, we crossed the *dMcm10* knockdown fly with enhancer trap lines P82 (inserted in *deadpan*) and B38 (inserted in *klingon*). P82 and B38 specifically express β-galactosidase in the photoreceptor cells of R3/R4/R7 and R7, respectively. The eye imaginal discs of third instar larvae were immunostained with anti-β-galactosidase antibody. R3/R4/R7 signals were detected in the eye discs of P82 control flies carrying *GMR*-GAL4>UAS-*GFPIR* (Figure 9A). However, knockdown of *dMcm10* caused a significant reduction in the number of R7 signals while the R3/R4 signals were unaffected (Figure 9B). Similarly, R7 signals dramatically reduced in the eye discs in the *dMcm10* knockdown with B38 flies (Figure 9E) compared to the control flies (Figure 9D). A different *dMcm10* knockdown fly line targeting a distinct region of dMcm10 (aa3 to aa117) were used to rule out the possibility of off-target effect of the RNAi. These also showed similar reduction of the R7 photoreceptor cells in the eye discs (Figure 9C and 9F). The R7 cell differentiation defect in *dMcm10* knockdown flies was further confirmed using another marker for R7, the anti-Prospero antibody (Figure 10). Using this method we also observed a strong reduction in the number of R7 photoreceptors on knockdown of *dMcm10* in eye imaginal discs (Figure 10D) compared with the controls (Figure 10A). These observations give further confirmation that knockdown of *dMcm10* specifically inhibits the differentiation of R7 photoreceptor cells.

### Knockdown of *dMcm10* inhibits R7 photoreceptor cell differentiation independently of its apoptotic effect

Since the knockdown of *dMcm10* induces apoptosis, it is possible that the differentiation defect was caused by the pro-apoptotic effect of the *dMcm10* knockdown. To address this, we analyzed R7 cell differentiation in *dMcm10* knockdown eye imaginal discs with insufficient apoptosis using an R7 photoreceptor cell marker, anti-Prospero antibody, and an apoptosis marker, anti-cleaved Caspase-3 antibody. In the control flies, R7 cells differentiate normally in the posterior region (Figure 10A), and no apoptotic cells were detected (Figure 10B). However, the numbers of R7 photoreceptor cells were strongly reduced in the *Mcm10* knockdown flies (Figure 10D) and in flies where *dMcm10* knockdown was combined with P35 expression (Figure 10G). In the *dMcm10* knockdown flies, many apoptotic signals appeared in the posterior region, showing that knockdown of *dMcm10* induces apoptosis (Figure 10E). Although cell death was inhibited by expression of P35 (Figure 10H), R7 cell differentiation was not rescued in *the* eye discs (Figure 10G). These data suggest that *dMcm10* depletion causes the R7 photoreceptor cell differentiation defect independently of its apoptosis-inducing effect.

## Discussion

Although Mcm10 was initially identified as a protein with a role in DNA replication, functions of Mcm10 in different cellular processes have been suggested [17], [23], [24]. In this study, we characterized *Drosophila* Mcm10 during development of the compound eye. We observed a delay in the S-phase in eye imaginal discs of *dMcm10* knockdown flies, indicating that dMcm10 plays a role in DNA replication. These observations are consistent with studies with *Drosophila* Mcm10 hypomorphic allele demonstrating S-phase delay in larval brain and an under-replication phenotype of salivary gland polytene chromosomes [24].

We also demonstrated that the delay in S-phase progression was accompanied by a delayed entry into and progress across M phase in *dMcm10* knockdown eye discs. Consistent with this, it was reported that depletion of *dMcm10* by RNAi in *Drosophila* cultured cells results in undercondensed metaphase chromosomes [23]. Asynchronous progression of M phase and anaphase bridges in early embryo nuclei and defects in chromosome decondensation in nurse cell nuclei were also observed with the *Drosophila Mcm10^Scim19^* allele [24].

The defects in the S and M phases of the cell cycle could result in genomic DNA damage. In fact, our studies revealed that the depletion of *dMcm10* in the eye discs caused an increase in the level of chromatin bound H2AvD suggesting that knockdown of *dMcm10* causes DNA instability. Recently, a direct interaction has been reported between human Mcm10 and RecQ4 which is also thought to be important for genomic DNA stability [34]. Induction of apoptosis can also be a consequence of DNA damage in cells and in fact a substantially high number of caspase-3 positive cells were detected in third instar larval eye imaginal discs of *dMcm10* knockdown flies. Furthermore, co-expression of baculovirus P35, an apoptotic inhibitor, in the *dMcm10* knockdown flies could suppress cell death. These observations indicate that knockdown of *dMcm10* induces caspase-dependent apoptosis in eye imaginal discs. This data is consistent with another study in human cells showing that Mcm10 depletion causes apoptosis [35].

Mcm10 plays important roles in DNA replication, chromosome condensation and heterochromatin formation [24]. In addition to these roles, we found that dMcm10 also participated in the differentiation of R7 photoreceptor cells during *Drosophila* eye development. Although we showed that its involvement in this process is independent of the apoptosis induced by *dMcm10* knockdown in eye discs, the exact mechanism by which dMcm10 affects R7 photoreceptor cell differentiation remains to be determined. One possibility is that dMcm10 may interact with chromatin regulators to regulate transcription of genes involved in R7 differentiation. Alternatively the effects of *dMcm10* knockdown on eye differentiation could be indirect, through incomplete DNA replication causing a delay of cell cycle progression. Further analysis, in particular studies looking at the genetic interactions between Mcm10 and other candidate genes should clarify these points and define the part played by dMcm10 in eye development.

## Supporting Information

Figure S1 **Specificity of anti-dMcm10 rabbit polyclonal antibody.** Western immunoblot analysis. Protein extracts were prepared from third instar larvae with the following genotypes: *yw*, +, *Act5C*-GAL4/UAS-*HA-dMcm10* (lane 1); Canton S (lane 2); *yw*, +, *Act5C*-GAL4/UAS-*dMcm10IR* (lane 3). The blots were probed with anti-dMcm10 antibodies (lane 1, 2, and 3). Anti-α-tubulin was used as a loading control. White arrowhead shows the position of dMcm10. M indicates marker.
(TIF)

Figure S2 **Two different** ***dMcm10*** **knockdown lines induced a similar delay in S phase in eye imaginal discs.** The eye imaginal discs were labeled with EdU (Red). (A) *GMR*-GAL4/+; +; +; (B) *w*; +; UAS-*Mcm10IR_633-700_*; (C) *w*; UAS-*Mcm10IR_3-117_*; +; (D) *GMR*-GAL4/+; +; UAS-*Mcm10IR_633-700_*/UAS-*Mcm10IR_633-700_*; (E) *GMR*-GAL4/+; UAS-*Mcm10IR_3-117_*/UAS-*Mcm10IR_3-117_*; +. The arrows show the positions measured. The bars indicate 10 μm. The flies were reared at 28°C. White arrowheads indicate morphogenetic furrow (MF). (a) indicates anterior, (p) indicates posterior.
(TIF)

Table S1 **Summary of effects of expression of** ***dMcm10*** **dsRNA with several GAL4 driver lines.**
(DOCX)

## References

[1] Wohlschlegel JA, Dhar SK, Prokhorova TA, Dutta A, Walter JC (2002) Xenopus Mcm10 binds to origins of DNA replication after Mcm2-7 and stimulates origin binding of Cdc45. Mol Cell 9: 233–240. 11864598 10.1016/s1097-2765(02)00456-2

[2] Ricke RM, Bielinsky AK (2004) Mcm10 regulates the stability and chromatin association of DNA polymerase-alpha. Mol Cell 16: 173–185. 15494305 10.1016/j.molcel.2004.09.017

[3] Gregan J, Lindner K, Brimage L, Franklin R, Namdar M, et al (2003) Fission yeast Cdc23/Mcm10 functions after pre-replicative complex formation to promote Cdc45 chromatin binding. Mol Biol Cell 14: 3876–3887. 12972571 10.1091/mbc.E03-02-0090 PMC196582

[4] Heller RC, Kang S, Lam WM, Chen S, Chan CS, et al (2011) Eukaryotic origin-dependent DNA replication in vitro reveals sequential action of DDK and S-CDK kinases. Cell 146: 80–91. 21729781 10.1016/j.cell.2011.06.012 PMC3204357

[5] Kanke M, Kodama Y, Takakashi TS, Nakagawa T, Masukata H (2012) Mcm10 plays an essential role in origin DNA unwinding after loading of the CMG components. EMBO J 31: 2182–2194. 22433840 10.1038/emboj.2012.68 PMC3343466

[6] Izumi M, Yatagai F, Hanaoka F (2004) Localization of human Mcm10 is spatially and temporally regulated during the S phase. J Biol Chem 279: 32569–32577. 15136575 10.1074/jbc.M314017200

[7] Yang X, Gregan J, Lindner K, Young H, Kearsey SE (2005) Nuclear distribution and chromatin association of DNA polymerase alpha-primase is affected by TEV protease cleavage of Cdc23 (Mcm10) in fission yeast. BMC Mol Biol 6: 13. 15941470 10.1186/1471-2199-6-13 PMC1182370

[8] van Deursen F, Sengupta S, De Piccoli G, Sanchez-Diaz A, Labib K (2012) Mcm10 associates with the loaded DNA helicase at replication origins and defines a novel step in its activation. EMBO J 31: 2195–2206. 22433841 10.1038/emboj.2012.69 PMC3343467

[9] Warren EM, Vaithiyalingam S, Haworth J, Greer B, Bielinsky AK, et al (2008) Structural basis for DNA binding by replication initiator Mcm10. Structure 16: 1892–1901. 19081065 10.1016/j.str.2008.10.005 PMC2636851

[10] Du W, Stauffer ME, Eichman BF (2012) Structural biology of replication initiation factor Mcm10. Subcell Biochem 62: 197–216. 22918587 10.1007/978-94-007-4572-8_11 PMC5023279

[11] Du W, Josephrajan A, Adhikary S, Bowles T, Bielinsky AK, et al (2013) Mcm10 self-association is mediated by an N-terminal coiled-coil domain. PLoS One 8: e70518. 23894664 10.1371/journal.pone.0070518 PMC3720919

[12] Robertson PD, Warren EM, Zhang H, Friedman DB, Lary JW, et al (2008) Domain architecture and biochemical characterization of vertebrate Mcm10. J Biol Chem 283: 3338–3348. 18065420 10.1074/jbc.M706267200 PMC2753450

[13] Cook CR, Kung G, Peterson FC, Volkman BF, Lei M (2003) A novel zinc finger is required for Mcm10 homocomplex assembly. J Biol Chem 278: 36051–36058. 12844493 10.1074/jbc.M306049200

[14] Ricke RM, Bielinsky AK (2006) A conserved Hsp10-like domain in Mcm10 is required to stabilize the catalytic subunit of DNA polymerase-alpha in budding yeast. J Biol Chem 281: 18414–18425. 16675460 10.1074/jbc.M513551200

[15] Warren EM, Huang H, Fanning E, Chazin WJ, Eichman BF (2009) Physical interactions between Mcm10, DNA, and DNA polymerase alpha. J Biol Chem 284: 24662–24672. 19608746 10.1074/jbc.M109.020438 PMC2782055

[16] Robertson PD, Chagot B, Chazin WJ, Eichman BF (2010) Solution NMR structure of the C-terminal DNA binding domain of Mcm10 reveals a conserved MCM motif. J Biol Chem 285: 22942–22949. 20489205 10.1074/jbc.M110.131276 PMC2906287

[17] Douglas NL, Dozier SK, Donato JJ (2005) Dual roles for Mcm10 in DNA replication initiation and silencing at the mating-type loci. Mol Biol Rep 32: 197–204. 16328881 10.1007/s11033-005-2312-x

[18] Liachko I, Tye BK (2005) Mcm10 is required for the maintenance of transcriptional silencing in Saccharomyces cerevisiae. Genetics 171: 503–515. 16085704 10.1534/genetics.105.042333 PMC1456767

[19] Liachko I, Tye BK (2009) Mcm10 mediates the interaction between DNA replication and silencing machineries. Genetics 181: 379–391. 19064704 10.1534/genetics.108.099101 PMC2644934

[20] Thu YM, Bielinsky AK (2013) Enigmatic roles of Mcm10 in DNA replication. Trends Biochem Sci 38: 184–194. 23332289 10.1016/j.tibs.2012.12.003 PMC3608807

[21] Aparicio JG, Viggiani CJ, Gibson DG, Aparicio OM (2004) The Rpd3-Sin3 histone deacetylase regulates replication timing and enables intra-S origin control in Saccharomyces cerevisiae. Mol Cell Biol 24: 4769–4780. 15143171 10.1128/MCB.24.11.4769-4780.2004 PMC416400

[22] Knott SR, Viggiani CJ, Tavaré S, Apricio OM (2009) Genome-wide replication profiles indicate an expansive role for Rpd3L in regulating replication initiation timing or efficiency, and reveal genomic loci of Rpd3 function in Saccharomyces cerevisiae. Genes Dev 23: 1077–1090. 19417103 10.1101/gad.1784309 PMC2682954

[23] Christensen TW, Tye BK (2003) Drosophila MCM10 interacts with members of the prereplication complex and is required for proper chromosome condensation. Mol Biol Cell 14: 2206–2215. 12808023 10.1091/mbc.E02-11-0706 PMC194871

[24] Apger J, Reubens M, Henderson L, Gouge CA, IIic N, et al (2010) Multiple functions for Drosophila Mcm10 suggested through analysis of two Mcm10 mutant alleles. Genetics 185: 1151–1165. 20498296 10.1534/genetics.110.117234 PMC2927746

[25] Wolff T, Ready DF (1993) Pattern formation in the Drosophila retina. In: Michael Bate, editor. The Development of Drosophila melanogaster. pp. 1277–1326.

[26] Hirose F, Ohshima N, Shiraki M, Inoue YH, Taguchi O, et al (2001) Ectopic expression of DREF induces DNA synthesis, apoptosis, and unusual morphogenesis in the Drosophila eye imaginal disc: possible interaction with Polycomb and trithorax group proteins. Mol Cell Biol 21: 7231–7242. 11585906 10.1128/MCB.21.21.7231-7242.2001 PMC99898

[27] Reichhart JM, Ligozygakis P, Naitza S, Woerfel G, Imler JL, et al (2002) Splice-activated UAS hairpin vector gives complete RNAi knockout of single or double target transcripts in Drosophila melanogaster. Genesis 34: 160–164. 12324974 10.1002/gene.10122

[28] Hayashi Y, Kato M, Seto H, Yamaguchi M (2006) Drosophila distal-less negatively regulates dDREF by inhibiting its DNA binding activity. Biochim Biophys Acta 1759: 359–366. 16949685 10.1016/j.bbaexp.2006.07.002

[29] Spradling AC (1986) P element-mediated transformation. In: Roberts DB, editor. Drosophila: A Practical Approach. pp. 175–197

[30] Robertson HM, Preston CR, Phillis RW, Johnson-Schlitz DM, Benz WK, et al (1988) A stable genomic source of P element transposase in Drosophila melanogaster. Genetics 118: 461–470. 2835286 10.1093/genetics/118.3.461 PMC1203300

[31] Yamaguchi M, Hirose F, Nishimoto Y, Naruge T, Ikeda M, et al (1995) Expression patterns of DNA replication enzymes and the regulatory factor DREF during Drosophila development analyzed with specific antibodies. Biol Cell 85: 147–155. 8785516 10.1016/0248-4900(96)85275-0

[32] Sun J, Tower J (1999) FLP recombinase-mediated induction of Cu/Zn-superoxide dismutase transgene expression can extend the life span of adult Drosophila melanogaster flies. Mol Cell Biol 19: 216–228. 9858546 10.1128/mcb.19.1.216 PMC83880

[33] Zhu W, Ukomadu C, Jha S, Senga T, Dhar SK, et al (2007) Mcm10 and And-1/CTF4 recruit DNA polymerase alpha to chromatin for initiation of DNA replication. Genes Dev 21: 2288–2299. 17761813 10.1101/gad.1585607 PMC1973143

[34] Xu X, Rochette PJ, Feyissa EA, Su Tv, Liu Y (2009) MCM10 mediates RECQ4 association with MCM2-7 helicase complex during DNA replication. EMBO J 28: 3005–3014. 19696745 10.1038/emboj.2009.235 PMC2760112

[35] Chattopadhyay S, Bielinsky AK (2007) Human Mcm10 regulates the catalytic subunit of DNA polymerase-alpha and prevents DNA damage during replication. Mol Biol Cell 18: 4085–4095. 17699597 10.1091/mbc.E06-12-1148 PMC1995709

