# Supplementary figures and images for: Drosophila Mcm10 Is Required for DNA Replication and Differentiation in the Compound Eye
Source: PLoS One. 2014 Mar 31;9(3):e93450. doi: 10.1371/journal.pone.0093450 (PMC3970972; doi:10.1371/journal.pone.0093450)

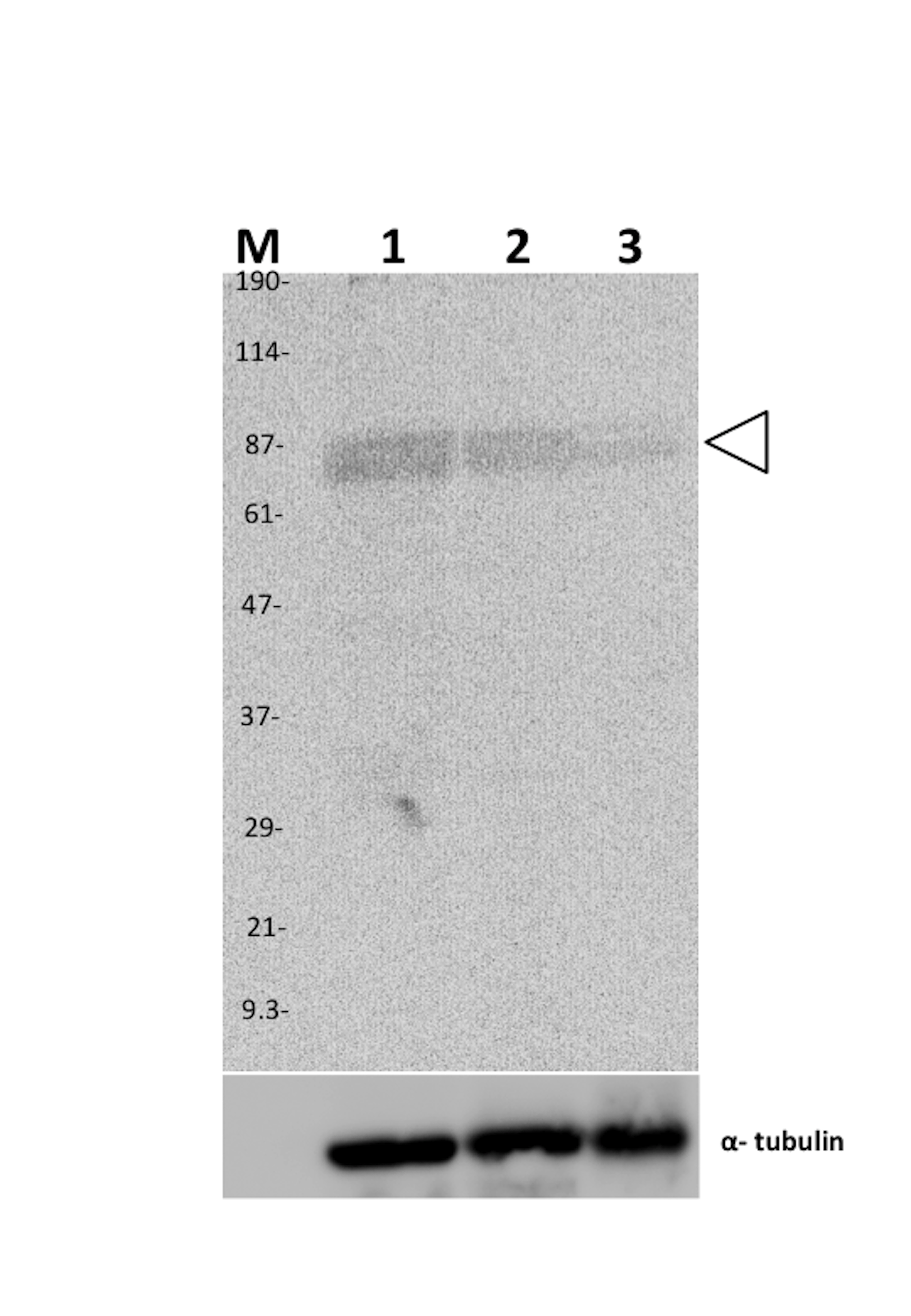

Supplement: Figure S1 — Specificity of anti-dMcm10 rabbit polyclonal antibody. Western immunoblot analysis. Protein extracts were prepared from third instar larvae with the following genotypes: yw, +, Act5C-GAL4/UAS-HA-dMcm10 (lane 1); Canton S (lane 2); yw, +, Act5C-GAL4/UAS-dMcm10IR (lane 3). The blots were probed with anti-dMcm10 antibodies (lane 1, 2, and 3). Anti-α-tubulin was used as a loading control. White arrowhead shows the position of dMcm10. M indicates marker. (TIF) [file pone.0093450.s001.tif]
