# Supplementary material for: Drosophila Mcm10 Is Required for DNA Replication and Differentiation in the Compound Eye
Source: PLoS One. 2014 Mar 31;9(3):e93450. doi: 10.1371/journal.pone.0093450 (PMC3970972; doi:10.1371/journal.pone.0093450)
Supplement: Table S1 — Summary of effects of expression of dMcm10 dsRNA with several GAL4 driver lines. (DOCX) [file pone.0093450.s003.docx]

**Table S1**

**Summary of effects of expression of dMcm10 dsRNA with several GAL4 driver lines**

| **GAL4 driver** | **Chromosome linkage** | **Expression pattern** | **Phenotype** |
| --- | --- | --- | --- |
| ***GMR-*GAL4** | X | Eye | Small and rough eye |
| ***Sg-*GAL4** | X | Salivary gland | Small salivary gland |
| ***En-*GAL4** | II | Wing | N.D. |
| ***Act5C-*GAL4** | III | Whole body | Pupa lethal |

**ND: No detectable phenotype**
